# RNA Surveillance by the Nuclear RNA Exosome: Mechanisms and Significance

**DOI:** 10.3390/ncrna4010008

**Published:** 2018-03-11

**Authors:** Koichi Ogami, Yaqiong Chen, James L. Manley

**Affiliations:** 1 Department of Biological Chemistry, Graduate School of Pharmaceutical Sciences, Nagoya City University, Nagoya 467-8603, Japan; 2 Department of Biological Sciences, Columbia University, New York, NY 10027, USA; yc2906@columbia.edu (Y.C.); jlm2@columbia.edu (J.L.M.)

**Keywords:** exosome, RNA surveillance, RNA processing, RNA degradation

## Abstract

The nuclear RNA exosome is an essential and versatile machinery that regulates maturation and degradation of a huge plethora of RNA species. The past two decades have witnessed remarkable progress in understanding the whole picture of its RNA substrates and the structural basis of its functions. In addition to the exosome itself, recent studies focusing on associated co-factors have been elucidating how the exosome is directed towards specific substrates. Moreover, it has been gradually realized that loss-of-function of exosome subunits affect multiple biological processes, such as the DNA damage response, R-loop resolution, maintenance of genome integrity, RNA export, translation, and cell differentiation. In this review, we summarize the current knowledge of the mechanisms of nuclear exosome-mediated RNA metabolism and discuss their physiological significance.

## 1. Introduction

**Figure 1:**
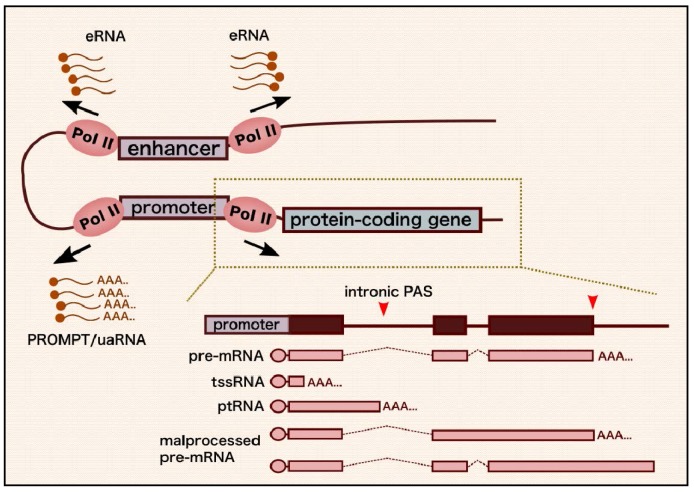
Schematic depiction of Polymerase II transcripts generated from enhancers and gene promoters. Both enhancers and promoters are transcribed bi-directionally and produce various types of transcripts, including messenger RNA precursors (pre-mRNA), transcription start site-associated RNA (tssRNA), prematurely terminated RNA (ptRNA), upstream antisense RNA (uaRNA) or promoter upstream transcript (PROMPT), and enhancer RNA (eRNA). The exosome functions in nuclear RNA surveillance to degrade these RNAs, as well as misprocessed messenger RNA (mRNA) precursors, such as intron-retained and poly(A) signal-mediated cleavage, and polyadenylation-defective pre-mRNAs.

Regulation of RNA maturation and degradation is a crucial step in gene expression. The nuclear RNA exosome has a central role in monitoring nearly every type of transcript produced by RNA polymerase I, II, and III (Pol I, II, and III). The exosome guarantees fidelity of the mature 3′ ends of certain stable RNA species, such as ribosomal RNAs (rRNAs), transfer RNAs (tRNAs), telomeric RNAs, small nuclear and nucleolar RNAs (snRNAs and snoRNAs), not only by catalyzing 3′ end trimming, but also by degrading transcripts containing an incomplete 3′ end [1,2,3]. Besides, processing of messenger RNA precursors (pre-mRNAs), such as by splicing and 3′ end formation, is also under the surveillance of the exosome (Figure 1) [4,5,6,7,8,9,10,11,12,13]. 

Intriguingly, recent advances in RNA sequencing techniques have enabled detection of novel Pol II transcripts (Figure 1), which are expressed at extremely low levels because of rapid RNA turnover by the exosome. A large fraction of these RNAs can be categorized as long non-coding RNA (lncRNA). The most well-known lncRNA substrates for the exosome are cryptic unstable transcripts (CUTs) in yeast [14,15,16], and their human counterparts, promoter upstream transcripts (PROMPTs) or upstream antisense RNAs (uaRNAs) [17,18], which arise due to antisense transcription from divergent gene promoters. The exosome removes sense non-coding transcripts, such as prematurely terminated RNAs (ptRNAs) [19], which are prematurely terminated and polyadenylated at a poly(A) signal (PAS) typically located in an intron of a protein-coding gene [20], and transcription start site (TSS)-associated RNAs (tssRNAs), which are infrequent short non-coding RNAs (ncRNA) (20–65 nt) generated as a result of promoter-proximal termination of sense transcription [21]. Transcriptional enhancers are also transcribed bi-directionally, and produce a class of lncRNA called enhancer RNAs (eRNAs). It was reported that exosome-sensitive eRNAs emerge from virtually all active enhancer regions, determined by comprehensive cap analysis of gene expression (CAGE) analyses [22]. Furthermore, long intergenic RNAs (lincRNAs) are also exosome targets [23], although they are generally more stable than uaRNA and eRNA [24].

Strikingly, recent studies have been gradually revealing that the exosome is involved in multiple important biological processes. Those include the DNA damage response (DDR), R-loop resolution, maintenance of genome integrity, RNA export, translation, and cell differentiation. In this work, we review and update our current understanding regarding structural insights into RNA degradation by the exosome and its associated co-factors. We also summarize how abrogation of the functions of the exosome impacts cellular processes in mammals.

## 2. The Nuclear RNA Exosome: Structure and RNA Degradation Mechanisms

The eukaryotic nuclear RNA exosome is a 3′–5′ exonuclease complex, consisting of a 9-protein catalytically inactive core complex (EXO-9) and two catalytic subunits, Rrp6 (also known as PM/Scl-100 or EXOSC10), and Dis3 (also known as Rrp44 or EXOSC11). EXO-9 forms a double-layered barrel-like structure that comprises six ribonuclease (RNase) pleckstrin homology (PH)-like proteins (Rrp41, Rrp42, Rrp43, Rrp45, Rrp46, and Mtr3) and three S1/K homology (KH) “cap” proteins (Rrp4, Rrp40, and Csl4) [3]. The two catalytic subunits occupy opposite ends of EXO-9 to constitute EXO-11. Rrp6 is placed at the top of the S1/KH cap ring near the RNA entry pore, and Dis3 is tethered to the bottom of EXO-9 near the RNA exit pore [25,26,27]. Both Rrp6 and Dis3 are 3′–5′ exonucleases, but the latter also has endonucleolytic activity [28,29,30]. Rrp6 widens the central channel of core EXO-9 and allosterically stimulates Dis3 activity [26]. Recently, a study focused on the last 100 amino acids of Rrp6, referred to as a “lasso,” and revealed that the lasso binds RNA proximal to the EXO-9 channel and enhances RNA decay [31]. In humans, both Rrp6 and Dis3 are mostly nuclear, but Rrp6 shows significant nucleolar enrichment [32,33], whereas Dis3 is excluded from the nucleoli [33,34]. In contrast to humans, yeast Rrp6 is restricted to the nucleus, but Rrp6 and Dis3 are both present in the nucleoplasm and nucleolus [32,35].

**Figure 2:**
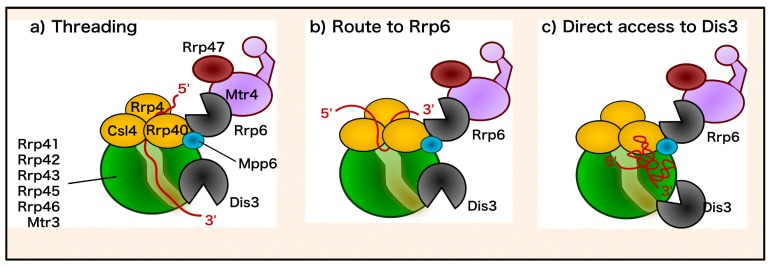
Structure of the RNA exosome and paths for RNA substrates to the catalytic subunits. (**a**) Threading route: RNA enters the central channel of the core exosome and reaches the active site of Dis3. (**b**) Route to Rrp6: RNA traverses the cap structure and reaches the active site of Rrp6. (**c**) Direct access to Dis3. RNA bypasses the central channel and directly accesses Dis3.

Three additional co-factors, Mtr4 (in humans, also known as SKIV2L2 or MTREX (nomenclature recently suggested by HUGO)), Rrp47 (also known as C1D), and Mpp6, are required for maximal activity of the nuclear exosome. Rrp47 interacts with Rrp6 to provide a binding platform for Mtr4, an essential DExH-box RNA helicase [36]. Mpp6 binds to the cap subunit Rrp40, and enhances Mtr4 helicase activity [37,38]. This activity is required to unwind secondary structures formed at the 3′ end of RNA substrates, so that the resultant single-stranded RNA substrates can be threaded into the central channel of the core complex in a 3′ to 5′ orientation [39]. Dis3 degrades RNAs threaded through the entire central channel (Figure 2a), whereas Rrp6 degrades or trims the RNA that enters into the S1/KH cap ring, and then traverses the cap to reach the Rrp6 active site (Figure 2b) [26,40,41]. In addition, there is an alternative path by which the RNA can directly access the Dis3 active site (Figure 2c) [42]. The RNA channeling, but not the direct route, induces a conformational change in Dis3 [42]. The estimated path lengths of the threading and direct access in vitro are ~30 nt and ~10 nt, respectively [42,43,44,45]. Recent studies in *Saccharomyces cerevisiae* have revealed that RNA substrates show preferences for a specific path to Dis3 [46,47]. Notably, identification of transcriptome-wide interactions of RNAs with individual exosome subunits using the ultraviolet (UV) crosslinking and analysis of cDNA (CRAC) technique in growing budding yeast cells showed that RNA substrates produced by all three RNA polymerases (Pol I, II, and III) exhibit preferences [47]. Interestingly, whichever the route is, Mtr4 is required for RNA degradation [47]. In addition to these two paths, a potential new route to Dis3 was recently suggested [48]: by assessing the average length of RNAs protected by the exosome in living budding yeast using CRAC analysis, it was found that there are not only ~10 nt (reflecting direct access) and 39 and 44 nt (likely reflecting RNAs threaded through the channel and also protected by co-factors) peaks, but also a ~20 nt broad peak that was not described in in vitro studies.

## 3. Molecular Apparatus for RNA Targeting of the Exosome in Yeasts and Humans

**Figure 3:**
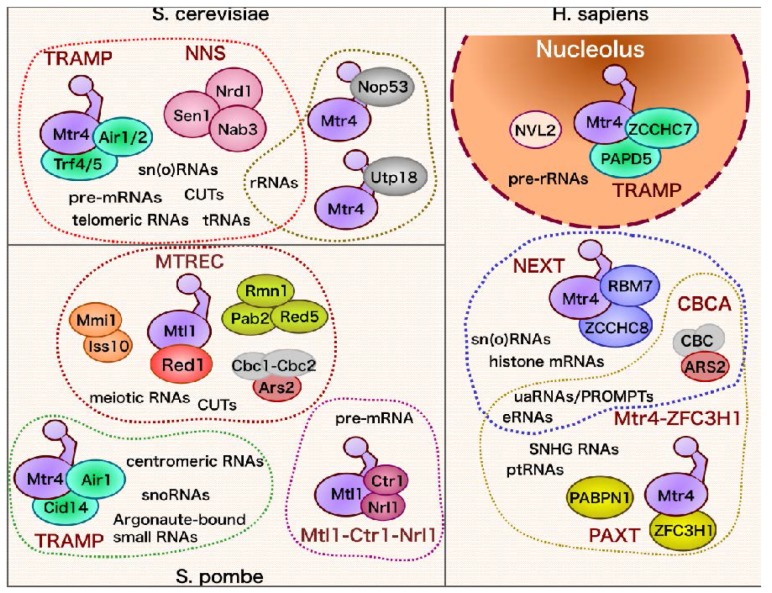
Overview of Mtr4-containing exosome adaptor complexes in yeasts and humans. The RNA helicase Mtr4 participates in multiple distinct exosome adaptor complexes to complete degradation and/or processing of specific RNA substrates. Mtr4-containing complexes identified in *Saccharomyces cerevisiae* (**upper-left**), *Schizosaccharomyces* *pombe* (**lower left**) and *Homo sapiens* (**right**) are shown.

The fact that the exosome targets a wide variety of transcripts raises an important question: how is the exosome specifically recruited to particular RNA substrates? Recent studies have identified a number of nuclear exosome–adaptor complexes, which help the exosome load onto selective RNAs [2,3,49]. The components of the adaptors are largely conserved, especially between fission yeast and humans (Table 1). Importantly, Mtr4 is contained in all of the adaptor complexes, indicating that Mtr4 is a central and essential factor for formation of the complexes and for their functions (Figure 3). 

### 3.1. Saccharomyces cerevisiae

The Trf4/5-Air1/2-Mtr4 polyadenylation complex (TRAMP) was first described in *S. cerevisiae*, and now is the most well-characterized co-factor that assists exosome-mediated RNA degradation and processing in budding yeast nuclei. Soon after recognizing the importance of polyadenylation of hypomodified methyonyl initiator transfer RNA (tRNA_i_^Met^) by the non-canonical poly(A) polymerase Trf4 for exosome-dependent tRNA quality control [50], the full composition of the responsible protein complex, TRAMP (Mtr4, Trf4, and the Zn-knuckle RNA-binding protein Air1 or Air2), was determined [14,51,52]. Later, another TRAMP complex containing Trf5, a close homolog of Trf4, was identified [53]. Air1/2 provides RNA-binding capability and is also critical for TRAMP assembly [54,55,56]. TRAMP recognizes a variety of transcripts [12], such as tRNAs [50,52,57,58,59], rRNAs [59,60,61], sn/snoRNAs [59,62,63], telomeric RNAs [64], CUTs [14,59,64], and pre-mRNAs [59,65,66,67], and these substrates are commonly polyadenylated by Trf4/5. In TRAMP, Mtr4 plays roles in RNA unwinding and modulation of poly(A)-tail length of RNA substrates [57,68,69,70,71,72,73]. Although TRAMP itself has an RNA-binding capacity, its efficient recruitment to RNA substrates is further assisted by the Nrd1–Nab3–Sen1 (NNS) complex [74]. Nrd1 and Nab3 are RNA-binding proteins that recognize specific sequence elements [59,75,76], whereas Sen1 has DNA/RNA helicase activity, which promotes dissociation of Pol II from the template DNA [77,78,79]. Importantly, NNS travels with a transcribing Pol II by interacting with the C-terminal domain of the Pol II largest subunit (CTD) and terminates transcription when the sequence elements emerge on the nascent RNAs [78,80,81,82,83,84,85]. NNS-dependent transcription termination is further promoted by the cleavage/polyadenylation factor Pcf11 [86]. Nrd1 interacts with the CTD-containing heptapeptide repeats (YSPTSPS) phosphorylated on Ser5 (Ser5P) through its CTD interaction domain (CID) [83,87,88]. The Nrd1 CID also binds to a CTD mimic motif in Trf4 [89]. The Nrd1 CID interacts with Trf4 and Pol II in a mutually exclusive manner, and therefore, NNS-mediated transcription termination and TRAMP/exosome-mediated RNA degradation are coordinated [89]. Notably, proteins homologous to the NNS components were found in *Schizosaccharomyces pombe* and humans (Table 1). Both *S. pombe* and humans have Sen1 homologs, Sen1 and Senataxin (SETX), respectively. *S. pombe* has the Nrd1 homolog Seb1 [90], which has Ser5P-CTD- and RNA-binding abilities [91,92]. However, although Seb1 is involved in transcription termination and alternative polyadenylation, no NNS-like function was observed [91,93,94]. Functions of the human CID-containing homolog of Nrd1, SCAF8 [95], remain unexplored, except that SCAF8 can bind to the elongating phosphorylated CTD [96,97]. Also, human RALY protein is somewhat similar to Nab3; the RNA recognition motif (RRM) in RALY shares 31% amino acid identity with the Nab3 RRM [98]. However, there is currently no evidence that these putative homologs of NNS subunits form an NNS-like complex and regulate human TRAMP functions.

Several other exosome partners exist in budding yeast. Utp18 and Nop53, an early and late associating small subunit processome factor, respectively, were shown to interact with the exosome to regulate ribosomal RNA precursor (pre-rRNA) processing [99]. Both proteins contain a conserved motif termed an arch-interacting motif (AIM), which directly dock to the arch domain of Mtr4. Recent X-ray crystallography and NMR analyses revealed the structural basis of Mtr4–Nop53 interaction and showed that the Mtr4 arch can bind Nop53 and RNA simultaneously [100]. The G-patch protein Sqs1/Pfa1 also contains a perfect AIM consensus sequence, and thus binds to the Mtr4 arch domain; however, the roles of the interaction remain elusive [99]. In addition, Babour et al. reported that the chromatin remodeling complex ISW1 physically interacts with the exosome in an RNase-insensitive manner [101]. Interestingly, this interaction is enhanced in the export-incompetent thermo-sensitive *npl3-1* mutant strain. ISW1 is required to retain export-defective poly(A)-tailed RNAs on chromatin and remove them by recruiting the exosome. This finding implies that ISW1/exosome participates in a messenger ribonucleoprotein (mRNP) nuclear export surveillance system.

### 3.2. Schizosaccharomyces pombe

The fission yeast *S. pombe* has a complex similar to *S. cerevisiae* TRAMP, consisting of Mtr4, Air1 and the Trf4/5 family of poly(A) polymerase Cid14 [102]. It functions in heterochromatic gene silencing at centromeric repeats [102,103,104] and polyadenylation-dependent decay of centromeric RNAs [105,106], snoRNA precursors [107], and Argonaute-bound small RNAs [108]. The precise mechanism of TRAMP recruitment to target transcripts remains unclear; however, Mlo3, the *S. pombe* homolog of mRNA export factor Yra1 or ALYREF, was shown to interact with TRAMP to silence centromeric transcripts [103,104]. Besides, the THO complex, which coordinates the steps from transcription to RNA export, is required to maintain TRAMP at snoRNA genes, and these complexes cooperate in the control of snoRNA expression, thus linking transcription and nuclear surveillance machineries [107]. Notably, Yra1 physically associates with the THO complex in *S. cerevisiae* [109,110], and therefore, it is possible that both Mlo3 and the THO complex work in the same pathway for TRAMP-mediated RNA metabolism. 

*S. pombe* has a second Mtr4 homologue protein named Mtl1 (Mtr4-like protein 1), which is independent of TRAMP. Mtl1 interacts with the zinc-finger protein Red1 and various other proteins to form a complex called Mtl1–Red1 core (MTREC) or nuclear RNA silencing (NURS) [111,112]. MTREC interacts with the exosome, presumably through Red1 but not Mtl1 [113]. In agreement with this, Mtl1 lacks the N-terminal motif that mediates the interaction of Mtr4 with Rrp6 and Rrp41 [36]. MTREC further associates with several sub-modules such as Iss10-Mmi1, Red5-Pab2-Rmn1, Ars2-Cbc1-Cbc2, and the canonical poly(A) polymerase Pla1 [111,112,113]. All of these sub-modules can bind to MTREC simultaneously, forming a large 11 subunit complex [113]. However, since the sub-modules show different stoichiometry for MTREC-binding, there might be various forms of the MTREC complex. The sub-modules enable MTREC to direct specific RNA targets for exosome-mediated decay. The YTH protein Mmi1 is a well-characterized regulator of meiotic gene expression [114,115,116]. Mmi1 programs meiotic transcripts for co-transcriptional decay by recognizing repeats of a short nucleotide motif termed determinant of selective removal (DSR), which are found within introns in some target genes [116,117,118,119]. Iss10 is required for stable interaction between Mmi1 and Red1, and thus, involved in meiotic gene regulation [120]. Red5 and Pab2 contribute to degradation of meiotic mRNAs [121,122] and CUTs [113], whereas depletion of the associating factor Rmn1 does not affect the amount of either meiotic mRNA or CUTs [112,113]. The cap-associated complex Ars2–Cbc1–Cbc2 is responsible for efficient CUT degradation [113], which is reminiscent of the function of the human cap-binding complex (CBC)–ARS2 (CBCA) complex. The human CBCA complex is required for degradation of PROMPTs/uaRNAs [123], which are comparable to yeast CUTs [17] (discussed below).

Mtl1 also forms a Red1-independent protein complex with the *Caenorhabditis elegans* NRDE-2 homologue Nrl1 and the coiled-coil- and DUF4078 domain-containing protein Ctr1 [111,113]. The Mtl1–Ctr1–Nrl1 complex further associates with splicing factors, and is suggested to degrade unspliced pre-mRNA [111,113].

### 3.3. Homo sapiens

In addition to Mtr4, factors homologous to the yeast TRAMP subunits are present in humans; the closest orthologues of Air1/Air2 and Trf4/Trf5 are the zinc-knuckle protein ZCCHC7 and the non-canonical poly(A) polymerase PAPD5 (also known as Trf4-2), respectively. These three proteins form the TRAMP-like complex [124]. Functions of TRAMP-like are thought to be restricted to nucleoli under normal cellular conditions, due to the strict nucleolar localization of ZCCHC7 [124]. The other subunits Mtr4 and PAPD5 are restricted to the nucleus with nucleolar enrichment [124,125,126]. Interestingly, it was recently shown that viral infection induces cytoplasmic translocalization of ZCCHC7 and Mtr4 to facilitate exosome-mediated viral RNA decay in the cytoplasm [127]. It has been shown that PAPD5 is responsible for poly- or oligo-adenylation of nucleolar RNAs, such as snoRNAs [128] and aberrant pre-rRNA species [124,129], suggesting that polyadenylation assists RNA 3′ processing and/or degradation by TRAMP-like. Of note, PAPD5 has a close paralog, PAPD7 (also known as Trf4-1), that has been suggested to interact with ZCCHC7 [56]. However, roles of PAPD7 in TRAMP-like remain unclear; PAPD7 is excluded from nucleoli [126], and in agreement with this, PAPD7 is dispensable for polyadenylation of aberrant pre-rRNA species [129]. In addition, there is no evidence of an interaction between PAPD7 and Mtr4 in several independent proteomics analyses [20,124,130].

Human TRAMP-like interacts with several additional proteins. It has been shown that TRAMP-like-mediated pre-rRNA processing is modulated by the AAA-ATPase NVL2 [131,132] and its regulatory factor tryptophan-aspartic acid (WD) repeat-containing protein WDR74 [133,134,135]. Moreover, splicing factors such as U4/U6·U5 tri-snRNP subunits and hnRNPs are found to associate with TRAMP-like complex [20,124,130]. The function of the interaction with splicing factors awaits further investigation. The nucleolar exosome can interact with the double-stranded RNA-binding protein DGCR8, which is well known as a microprocessor subunit, to degrade mature snoRNAs and telomerase RNA (hTR) [136]. It is noteworthy that although the physical interaction between DGCR8 and TRAMP-like has not been reported, both snoRNAs and hTR are targeted by the TRAMP-like complex [136,137,138,139]. 

In the nucleoplasm, at least two distinct exosome adaptors are present. One is Mtr4–ZFC3H1 or poly(A) tail exosome targeting complex (PAXT), which brings the exosome to various kinds of lncRNAs, including snoRNA host gene (SNHG) transcripts, eRNAs [49], uaRNAs [20,49], and ptRNAs [20]. Another is nuclear exosome targeting complex (NEXT) [124], comprising Mtr4, the RNA binding protein RBM7 and the Zn-knuckle protein ZCCHC8, which degrades PROMPTs/uaRNAs [124], replication-dependent histone mRNAs [123], eRNAs [140], snRNAs [123,141], and snoRNAs [140]. Of note, ZFC3H1 is a close homolog of *S. pombe* Red1, and therefore, Mtr4–ZFC3H1 is the human MTREC. Although RNA substrates of Mtr4–ZFC3H1 and NEXT partly overlap, there are clear differences in their features; Mtr4–ZFC3H1 substrates are longer in RNA body size and have a long poly(A)-tail [20,49]. The precise molecular fundamentals of substrate recognition by Mtr4–ZFC3H1 await further characterization. However, Meola et al. suggested the transient and partially RNA-dependent interaction between Mtr4–ZFC3H1 and the nuclear poly(A)-binding protein PABPN1 [49]. It has been shown that PABPN1 promotes exosome-dependent decay of nuclear poly(A)-tailed transcripts [142,143,144]. PABPN1-mediated RNA decay is dependent on RNA polyadenylation, which requires the canonical poly(A) polymerases PAPα/γ, but not the TRAMP subunit PAPD5 [142,143,144], and is thus termed PABPN1- and PAPα/γ-mediated RNA decay pathway (PPD) [143]. Notably, subsets of the PABPN1 substrates overlap with those of Mtr4–ZFC3H1 [20,49]. Yet, the fact that co-depletion of Mtr4 and PABPN1 resulted in synergistic accumulation of target transcripts suggests that Mtr4–ZFC3H1 and PABPN1 may work in both the same and redundant pathways [144]. It will be interesting to investigate if and how Mtr4–ZFC3H1 participates in the PPD pathway. RNA recognition by NEXT involves the connection with the ARS2-associated cap-binding complex CBCA [123], U-rich RNA binding capacity of RBM7 [140,141], and possibly the pre-mRNA 3′ processing complex [145]. CBCA and NEXT further associate with the zinc-finger CCCH domain-containing protein ZC3H18 (also known as NHN1) [123,146], and this interaction is important for cap-proximal Pol II stalling, transcription termination, 3′ end formation, and RNA decay [123,146,147]. The significance of the interaction between NEXT and the pre-mRNA 3′ processing complex remains undetermined. 

A nucleoplasmic protein NRDE2, which is the homolog of *S. pombe* Nrl1, also interacts with Mtr4. However, in contrast to the *S. pombe* counterpart, it is unlikely that Mtr4/NRDE2 associates with the whole exosome, since analysis using size-exclusion chromatography-coupled mass spectrometry (MS) revealed that Mtr4/NRDE2 elutes around 440 kDa, which is smaller than the exosome/Mtr4 complex (>600 kDa) [20]. In agreement with this, our recent MS analysis of NRDE2-interacting proteins did not detect any exosome subunits [148].

## 4. Significance of the Nuclear RNA Exosome in Mammalian Biological Processes

Loss-of-function of the exosome due to mutation or depletion of its subunits and co-factors can cause alterations in various biological processes [2], and ultimately contribute to human disease, such as multiple myeloma [149,150,151]. Despite various interesting phenotypes in yeast, such as altered chromatin modifications in exosome-deficient cells, we restrict discussion here to evidence provided using mammalian cells.

### 4.1. DNA Damage Response

The activity of the nuclear exosome is altered during the cellular DDR. The change is triggered by phosphorylation of the NEXT subunit RBM7 by the stress-related kinase p38 MAPK/MK2 [152,153]. Phosphorylated RBM7 is bound by the phosphoserine-binding protein 14-3-3, and loses its RNA-binding ability, which consequently leads to stabilization and accumulation of NEXT substrates such as PROMPTs [152]. Interestingly, cells become hypersensitive to a DNA damaging reagent when RBM7 is depleted, and cells lacking RBM7 exhibit poor survival after drug treatment [152]. Although it is still largely unclear how these changes in the DDR affect cell physiology, there are interesting suggestions that a fraction of promoter-associated lncRNAs can modulate transcription of neighboring genes. For example, *cyclin D1* (CCND1) PROMPTs upregulated in response to DNA damage by ionizing irradiation provide a binding platform for the RNA-binding protein FUS/TLS. FUS/TLS recruited to the CCND1 promoter through PROMPTs represses the histone acetyltransferase activity of CBP/p300, which results in decreased CCND1 transcription [154]. However, it seems that PROMPT-mediated gene regulation is not widespread, since no correlation was observed between altered expression of the downstream gene and increased PROMPT levels in DNA damage or Rrp40 depletion [155]. This might possibly indicate that most PROMPTs lack sequence elements necessary for recruiting specific RNA-binding proteins, and the action of only a small fraction of PROMPTs may be required for the DDR.

### 4.2. R-Loop Resolution and Genomic Integrity

R loops are three-stranded structures composed of the nascent RNA hybridized with DNA template and the resultant displaced single-stranded DNA (ssDNA). R-loop resolution is a critical step to maintain genome integrity, since the displaced ssDNA is vulnerable to DNA damage [156,157,158,159]. Moreover, R loops are associated with human disease (reviewed in [160,161,162,163]). Intriguingly, multiple studies have reported the involvement of the exosome in R-loop resolution and genome integrity. In yeast, depletion of Rrp6 or Trf4 leads to R loop-mediated genomic instability and hyperrecombination [164,165], as well as accumulation of aberrant truncated RNA products released from an R loop [166]. These factors also promote the loading of ssDNA binding protein RPA to double-strand breaks (DSBs), and activate the checkpoint kinase Mec1/ATR, which facilitates the formation of continuous Rad51 filaments to initiate homologous recombination [167]. Strikingly, overexpression of RNase H, which removes R loops by digesting the RNA strand of RNA/DNA hybrids, dramatically rescued the rate of genome instability in TRAMP-depleted cells [168]. In human cells, the DNA/RNA helicase SETX (Senataxin), which plays a key role in R-loop resolution [169], directly interacts with the exosome subunit Rrp45 [170]. The interaction requires sumoylation of SETX, which interestingly, is blocked by certain *SETX* mutations in ataxia oculomotor apraxia 2 (AOA2) patients. It is speculated that SETX recruits the exosome to R loops to promote degradation of the RNA unwound and released by SETX, and thus prevents possible rehybridization and the resultant DNA damage.

Over the last decade, the concept has emerged that exosome-mediated R-loop prevention is a critical step in immunoglobulin class switch recombination (CSR) and somatic hypermutation (SHM) in B lymphocytes [171]. To initiate CSR and SHM, activation-induced cytidine deaminase (AID) deaminates cytidines on both template and non-template DNA strands of transcribing switch regions. However, the template DNA strand hybridized with a nascent transcript cannot be modified by AID because of limited access to the template strand. Basu et al. identified the core RNA exosome EXO-9 as a key factor that promotes AID access to the template strand in the context of RNA/DNA hybrids, and thus, CSR and SHM [172]. The interaction between AID and the RNA exosome is promoted by the E3 ubiquitin ligase NEDD4, which regulates clearance of Pol II from the immunoglobulin switch region [173]. In mouse B cells and embryonic stem cells (ESCs) containing a conditional inversion allele of *Exosc3* (Rrp40) or *Exosc10* (Rrp6), which allows conditional ablation of the exosome by drug treatment, loss of the exosome results in enhanced R-loop formation and genomic instability, due to an increase of ncRNAs associated with TSS and superenhancers [174,175,176]. More recently, it was shown that Mtr4 has an RNA/DNA hybrid unwinding activity, and Mt4-deficient B cells exhibited greater R-loop formation at the immunoglobulin heavy chain locus [177]. 

### 4.3. RNA Export and Translation

In addition to its role in NEXT loading to nascent transcript 5′ ends, the CBC is required to initiate nuclear RNA export by recruiting various proteins. The TREX mRNA export complex is recruited to the 5′ end of mRNAs through the export adaptor proteins ALYREF and THO associating with CBC [178,179,180]. While splicing enhances TREX recruitment [181], the interaction of ALYREF with the cap-binding protein CBP20 was shown to stimulate nuclear export of capped intronless mRNAs [180]. CBC-associating factor ZC3H18 can also enhance export of intronless mRNAs [182]. Recently, Fan et al. showed that Mtr4 competes with the export adaptor protein ALYREF for binding to ARS2, and thus inhibits nuclear export, providing an important checkpoint to prevent undesired transport of aberrant RNAs into the cytoplasm [183]. Intriguingly, CBCA (CBC–ARS2) and ZC3H18 are also found in the ZFC3H1 interactomes [49], suggesting that Mtr4–ZFC3H1 can also be recruited to CBCA assembled on the 5′ cap structure. Therefore, it is possible that both NEXT and Mtr4–ZFC3H1 can antagonize ALYREF binding to CBCA. This competition, as well as the rapid RNA degradation of poly(A)-tailed lncRNAs by Mtr4–ZFC3H1, is particularly important, since normally unstable lncRNAs are exported to the cytoplasm in cells lacking Mtr4–ZFC3H1 [20,184]. Of note, there is a link between RNA 3′ end cleavage/polyadenylation and export. Several 3′ cleavage and polyadenylation factors interact with RNA export factors. For example, Pcf11 directly interacts with the yeast homolog of ALYREF, Yra1 [185]; CFIm68 directly binds to the mRNA export receptor NXF1 [186]; CPSF100 and CFIm proteins associate with the THO subunit THOC5 [187,188]; and CstF64 and PABPN1 help ALYREF-binding to mRNA 3′ ends [189]. Therefore, effective recruitment of RNA export complex, including ALYREF, is mediated not only by CBC, but also the 3′ processing machinery and a poly(A)-tail. Recent remarkable progress in ribosome profiling technologies [190] has led to the realization that ribosome binding or even translation of lncRNAs is pervasive in mammals [191,192,193,194,195,196,197]. Concordantly, exported lncRNAs in Mtr4–ZFC3H1 deficient cells become ribosome-associated and likely translated. Because of the translatability of lncRNAs, as well as the more mRNA-like structures of Mtr4–ZFC3H1 substrates (presence of the cap and a poly(A)-tail) than those of NEXT substrates [49], the aberrantly exported Mtr4–ZFC3H1 substrates appear to overwhelm translation machinery and disrupt the quantitative balance between ribosomes and translatable RNAs, which leads to global reduction in heavy polysomes and translation [20,184]. 

Recently, Sinturel et al. reported intriguing findings that diurnal oscillations in liver mass and hepatocyte size are regulated by rhythmic changes in ribosome biogenesis, in which the nuclear exosome plays a role [198]. In this study, using mice, they demonstrated that these changes are controlled by feeding time: diurnal changes were observed only in mice fed during night and ad libitum, but not in day-fed mice. Importantly, they found that the number of ribosomes also exhibited diurnal fluctuations. In the active/dark phase, translation of ribosomal protein mRNAs was found to be significantly enhanced, and thus, protein synthesis rates increased, while in the resting/light phase, ribosomal protein synthesis was decreased, leading to an imbalance between ribosomal proteins and rRNAs. TRAMP functions to rebalance the amount of these factors by polyadenylating and degrading excess rRNAs in incomplete ribosomal subunits. These events contribute to a daily rhythm of mouse liver protein content.

### 4.4. Stem Cell Self-Renewal and Differentiation

Precise regulation of the activity and maintenance of the fidelity of gene expression is vital for stem cell self-renewal, differentiation, and development. Studies have suggested that the nuclear RNA exosome is essential for maintaining progenitor cell function and preventing premature differentiation. A defective exosome pathway can lead to aberrant accumulation of RNAs, among which are mRNAs encoding differentiation-specific transcription factors, and ultimately break the balance between proliferation and differentiation. For example, the nuclear exosome directly degrades *GRHL3* transcripts, which encode a transcription factor critical for epidermal differentiation [199]. Depletion of the exosome subunit Rrp45 (EXOSC9) leads to loss of progenitor cells from the basal epidermal layer and premature differentiation. More recently, Skamagki et al. suggested that the exosome plays an important role in maintaining pluripotent stem cell redox status in mice [200]. They found that the transcription factor ZSCAN10, which activates transcription of *EXOSC1*/*2*/*5* genes, is expressed at a low level in induced pluripotent stem cell clones generated from aged tissue donors, and the decreased expression of RNA exosome subunits causes the accumulation of AU-rich element-containing RNAs, including glutathione peroxidase 2 (Gpx2). Overexpression of GPX2 increases the reduced form of glutathione, thus scavenging glutathione-mediated reactive oxygen species, which consequently blunts the DDR and reduces apoptosis. Similar defects were observed following knockdown (KD) of exosome subunits EXOSC2 and/or EXOSC8 in ESCs. Mtr4 is also important in cell proliferation and differentiation. On the one hand, Mtr4 expression is highly upregulated when the self-renewal state of ESCs is induced by inhibitors of kinases, known as 2 inhibitors (2i) [201]. On the other hand, KD of Mtr4 resulted in moderate to severe mouse ESC death [202]. Additionally, depletion of Mtr4 impairs mitosis and induces cell differentiation in the murine cancer cell lines Neuro2A and P19 [203]. All the above indicates that levels of the exosome subunits correlates with cell differentiation. Indeed, Rrp4/Rrp40/Rrp42/Rrp45 (EXOSC2/3/7/9) expression is enriched in progenitor cells, but decreased upon epidermal differentiation in humans [199]. These observations strongly suggest that an abundance of the exosome is a critical prerequisite to maintain stem and progenitor cells in an undifferentiated state. 

### 4.5. Influenza A Virus (IAV) Ribogenesis and Infectivity

A recent study revealed the significance of the exosome in influenza A virus (IAV) ribogenesis and growth [204]. In this study, Rialdi et al. analyzed the proteome of viral polymerase complex-interacting proteins, and identified the core exosome subunits. Intriguingly, they found that viral polymerase activity is attenuated in cells transfected with siRNAs against exosome subunits and in patient-derived cells harboring an *EXOSC3* (Rrp40) mutation. Importantly, viral growth was suppressed in these cells, indicating the essential role of the exosome in viral biogenesis. NEXT-assisted exosome seems to be co-opted by the viral RNA polymerase, since similar results were obtained following RBM7 KD. Moreover, synthesis of host/viral chimeric transcripts generated as a result of “cap snatching”, in which initiation of viral transcription is primed using 5′ ends of host transcripts (cap with 10–20 downstream nucleotides), is decreased upon exosome-depletion. Collectively, these results suggest that the nuclear exosome coordinates with viral polymerase during the initial steps of viral transcription with Pol II at host promoters to enhance influenza A virus ribogenesis and infectivity. From the evolutionary point of view, viruses need to integrate their biological activities into hosts by recycling regulatory RNAs generated by hosts. The exosome, as the hub of RNA surveillance system, can be co-opted by viruses to facilitate the efficient formation of cellular/viral hybrid RNAs and cap-snatching.

## 5. Conclusions and Perspectives

The RNA exosome and its co-factors monitor the versatility and specificity of a huge variety of RNA substrates, and thus plays a crucial role in regulating the activity and maintaining the fidelity of gene expression. Numerous studies have revealed that an impaired RNA surveillance system can break RNA homeostasis, and thus cause detrimental consequences in multiple biological processes leading to human diseases (reviewed by Morton et al. [149]). However, there are still many unanswered questions about both the fundamental and the pathological mechanisms of the nuclear exosome: how are both specificity and versatility of RNA substrates guaranteed at the same time in the RNA surveillance system? What is the comprehensive mechanism of the nuclear exosome in multiple biological processes, including maintenance of genome integrity and cell differentiation? Deeper understanding of the complexities of the RNA surveillance system has the potential to lead to novel therapeutic remedies to fight human disease.

## Figures and Tables

**Table 1:** Conservation of exosome co-factors in yeasts and humans.

Complex	*Saccharomyces cerevisiae*	*Schizosaccharomyces pombe*	*Homo sapiens*
TRAMP	Mtr4	Mtr4	Mtr4/SKIV2L2/MTREX
	Air1, Air2	Air1	ZCCHC7
	Trf4, Trf5	Cid14	PAPD5, PAPD7
NNS	Nrd1	Seb1	SCAF4, SCAF8
	Nab3	Nab3	RALY, RALYL, hnRNPC, hnRNPCL1, hnRNPCL2, hnRNPCL3, hnRNPCL4
	Sen1	Sen1	SETX
MTREC NURS Mtr4/ZFC3H1 PAXT	Mtr4	Mtl1	Mtr4/SKIV2L2/MTREX
	-	Red1	ZFC3H1
	-	Iss10	-
	Pho92	Mmi1	YTHDF1, YTHDF2, YTHDF3
	Sto1	Cbc1	CBP80/NCBP1
	Cbc2	Cbc2	CBP20/NCBP2, NCBP2L
	-	Ars2/Pir2	ARS2/SRRT
	-	Red5	ZC3H3
	Sgn1/Rbp1/Rbp29	Pab2	PABPN1, PABPN1L
	-	Rmn1	RBM26, RBM27
	Pap1	Pla1	PAPOLA, PAPOLB, PAPOLG
Mtl1-Ctr1-Nrl1	Mtr4	Mtl1	Mtr4/SKIV2L2/MTREX
	-	Ctr1	CCDC174
	-	Nrl1	NRDE2
NEXT	Mtr4	Mtr4	Mtr4
	-	-	RBM7
	-	-	ZCCHC8
Other	Utp18	Utp18	UTP18
	Nop53	Rrp16	NOP53
	ISW1	-	SMARCA5
	Rix7	Rix7	NVL/NVL2
	Nsa1	Wdr74	WDR74
	-	-	DGCR8
